# Cloning and Functional Analysis of the Aldehyde Dehydrogenase Gene *VvALDH* in the IAA Synthesis Pathway of *Volvariella volvacea*

**DOI:** 10.3390/jof11110773

**Published:** 2025-10-27

**Authors:** Mingjuan Mao, Lijuan Hou, Lin Ma, Ning Jiang, Jinsheng Lin, Shaoxuan Qu, Huiping Li, Ping Xu, Di Liu, Wei Ji

**Affiliations:** 1 Jiangsu Key Laboratory for Horticultural Crop Genetic Improvement, Institute of Vegetable Crop, Jiangsu Academy of Agricultural Sciences, Nanjing 210014, China; maomj02@163.com (M.M.); malin1590@sina.com (L.M.); 20020005@jaas.ac.cn (N.J.); 19980012@jaas.ac.cn (J.L.); ququzhibao@163.com (S.Q.); lhp211@163.com (H.L.); pingxu4523@163.com (P.X.); 2 Department of Horticulture and Landscape Architecture, College of Agriculture, Yanbian University, Yanji 133002, China; liudi@ybu.edu.cn; 3 Lianyungang Academy of Agricultural Sciences, Lianyungang 222000, China; jiwei100500@163.com

**Keywords:** *Volvariella volvacea*, primordium, aldehyde dehydrogenase, IAA synthesis pathway, gene function

## Abstract

*Volvariella volvacea*, the Chinese mushroom, is a high-temperature grass-rot fungus with great production potential, yet its low yield limits industrial development. Exogenous sodium acetate (NaAc) has been shown to increase yield by promoting indole-3-acetic acid (IAA) synthesis during the primordium stage, but the underlying mechanism remains unclear. In this study, the aldehyde dehydrogenase gene *VvALDH*, highly expressed at the primordium stage, was cloned and functionally characterized. *VvALDH* encodes a 1509 bp cDNA with a conserved aldehyde dehydrogenase domain. Using Agrobacterium-mediated transformation, overexpression lines showed a 4.76-fold increase in *VvALDH* expression, accompanied by higher biomass (38%), yield (83%), and IAA content (34%), while RNAi lines showed opposite trends. These results demonstrate that *VvALDH* promotes IAA biosynthesis, enhances primordium differentiation, and increases yield. Further analysis revealed its involvement in multiple IAA biosynthetic pathways, including indolepyruvate, tryptamine, and tryptophan side-chain oxidase pathways. This work clarifies the molecular basis of NaAc-mediated yield improvement and provides a theoretical foundation for genetic and cultivation strategies in *V. volvacea*.

## 1. Introduction

*Volvariella volvacea*, also known as the Straw mushroom and South China mushroom [1], is a high-temperature edible mushroom with a distinctive flavor and aroma [2]. It is globally recognized as a source of high-quality protein and ranks among the most important mushroom species for export [3,4], with significant market potential. It is rich in protein, polysaccharides, vitamins, and minerals [5] and thus serves as an ideal food for consumption [6]. *V. volvacea* is a medicinal–edible fungus with notable antioxidant and anti-tumor properties, and it can effectively promotion human immunity [7]. However, the development of the *V. volvacea* industry has been relatively slow in recent years. Apart from the difficulty in preservation, issues such as severe strain degeneration, slow mycelial growth, reduced yield, or even failure to fruit have severely hindered the industry’s progress [8].

Sodium acetate (NaAc) application to *V. volvacea* has yielded remarkable results. Hou et al. [9] reported that exogenous NaAc significantly increased IAA content by 27.27% during the primordium stage of *V. volvacea*, promoting primordium differentiation and consequently enhancing yield by 16.25%. Spraying IAA also confirmed a significant 12.62% increase in *V. volvacea* yield. In the early stage, our research group, through transcriptome and expression profile analysis combined with qPCR-based gene expression quantification, identified that the gene *VvALDH*, which is involved in the IAA biosynthesis pathway, was significantly upregulated during the primordium stage [10]. The pH buffering effect of NaAc during microbial fermentation plays a crucial role in the growth of fungal mycelia. Xiao et al. [11] found that adding an appropriate amount of NaAc can promote the development of *Agaricus bisporus* mycelia, increase liquid culture biomass, and enhances factory yield. However, the metabolic pathways and molecular mechanisms through which NaAc increases endogenous IAA content and promotes primordium differentiation remain unclear.

**Figure 1:**
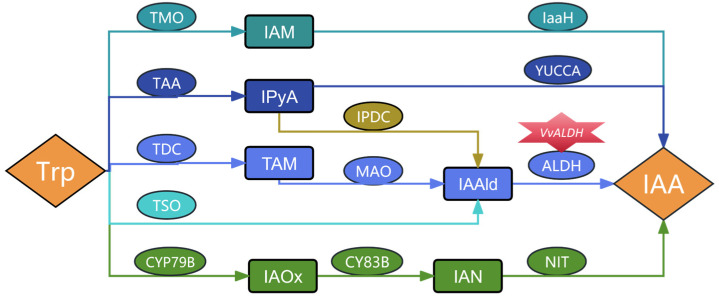
Tryptophan-dependent IAA synthesis pathway. Notes: Different colors represent different pathways, the oval shape is the enzyme, the rectangle is the intermediate product, and hexagonal stars are genes. Trp: tryptophan, IAM: indole-3-acetamide, IPyA: indole-3-pyruvate, IAAld: indole-3-acetaldehyde, TAM: tryptamine, IAOx: indole-3-acetaldehyde oxime, IAN: indole-3-acetonitrile, IAA: indole-3-acetic acid, TMO: tryptophan monooxygenase, IaaH: IAA amide hydrolase, TAA: tryptophan aminotransferase, IPDC: indole pyruvate decarboxylase, ALDH: aldehyde dehydrogenase, TDC: tryptophan decarboxylase, MAO: tryptamine oxidase. CYP79B: cytochrome P450 isoenzyme, CY83B: cytochrome P450 monooxygenase, NIT: nitrilase, TSO: tryptophan side chain oxidase, YUCCA: flavin monooxygenase.

IAA biosynthesis is divided into five pathways based on its main intermediate products: the indole-3-acetamide pathway (IAM), indole-3-pyruvic acid pathway (IPyA), tryptamine pathway (TAM), indole-3-acetaldoxime pathway (IAOx), and the tryptophan side-chain oxidase pathway (TSO) [12]. Pathways involving indole-3-acetaldehyde (IAAld) include the TAM, IPyA and TSO pathways. Additionally, studies on the involvement of endogenous IAA and IAAld in IAA biosynthetic pathways in edible fungi have been documented (Figure 1). Chen [13] reported that the stipe growth during different developmental stages of *V. volvacea* involves the IAM, TAM, and IAOx pathways of IAA synthesis. High-temperature stress modulates the expression of multiple key genes in the tryptophan (Trp) and IAA metabolic pathways of *Lentinula edodes* (*L. edodes*). Mycelial heat tolerance is primarily associated with intracellular IAA content, in which IAA is synthesized by the TAM-IAAld-IAA pathway [14]. Zhou et al. [15] reported that optimal concentrations of IAA promote biomass accumulation in *L. edodes*, while alleviating heat stress. In Trp-dependent pathways of microorganisms, three pathways lead to the formation of a common intermediate IAAld, which is oxidized to IAA by ALDH [16]. Fungal ALDHs are involved in IAA biosynthesis, regulating strain growth, development, and stress responses [17]. In summary, IAA impacts on the growth and development of edible fungi by modulating key processes: mycelial growth rate, biomass accumulation, stipe morphogenesis, and stress responses. As key enzymes in the IAA biosynthesis pathway, ALDHs also play an important role in yield improvement. Furthermore, ATMT facilitates targeted trait modification in edible fungal strains, including *L. edodes* [18], *Ganoderma lucidum* [19], and *Coprinopsis cinerea* [20], and with the successful development of engineered strains that exhibit enhanced disease resistance and high yields of secondary metabolites [21].

This study aims to elucidate the molecular regulatory mechanisms underlying the IAA biosynthesis pathway during the growth of *V. volvacea*. Currently, the mechanism of endogenous IAA biosynthesis remains unclear; most studies have focused on the effects of exogenous IAA application, whereas strain-specific research has advanced slowly and garnered limited attention. Given the diverse functions of IAA, including growth promotion, exploring the IAA biosynthetic pathways involved in *V. volvacea* development is of paramount importance. Functional analysis of the *VvALDH* gene was conducted to investigate the molecular mechanism by which NaAc affects *VvALDH* and thereby promotes IAA biosynthesis and primordium differentiation, providing a theoretical foundation for yield improvement.

## 2. Materials and Methods

The *V. volvacea* strain (V9715) is maintained by the Edible Fungi Innovation Team of Jiangsu Academy of Agricultural Sciences. *E. coli* strain DH5α competent cells and *Agrobacterium* tumefaciens GV3101 competent cells were obtained from Beijing Qingke Biotech Co., Ltd. (Beijing, China); the pMD™ 18-T Vector Cloning Kit was purchased from Takara (Beijing, China); and the pCAMBIA-1301 vector is maintained in our laboratory.

### 2.1. Medium

CYM liquid medium (per liter): glucose 20 g, maltose 10 g, peptone 2 g, yeast extract 2 g, MgSO_4_·7H_2_O 0.5 g, KH_2_PO_4_ 4.6 g. LB medium (per liter): yeast extract 5 g, tryptone 10 g, NaCl 10 g. Formulations for IM and Co-IM media refer to Lu [22]. Screening medium: PDA medium supplemented with cefotaxime (Cef, 200 μg/mL) and hygromycin (Hyg, 50 μg/mL).

### 2.2. Cloning and Bioinformatics Analysis of VvALDH

Primers were designed based on transcriptomic and genomic data of *V. volvacea* (sequences in Table 1). Genomic DNA (gDNA) and RNA were extracted, and cDNA was synthesized using the PrimeScript™ IV 1st Strand cDNA Synthesis Mix (Takara). The target fragment was recovered, purified, and ligated into the pMD18-T vector as described by Sun [23]; the recombinant vector was verified by colony PCR and sequenced by Beijing Qingke Biotechnology Co., Ltd.

Bioinformatics analyses included: identifying the open reading frame (ORF) and amino acid sequence using ORF Finder; locating introns and exons by comparing cDNA and gDNA sequences using GSDS 2.0; analyzing conserved domains by the NCBI Conserved Domain Database (CDD); predicting protein physicochemical properties using ExPASy ProtParam; predicting phosphorylation sites with NetPhos 3.1; and predicting secondary and tertiary structures using PRABI-GERLAND and SWISS-MODEL [24]. Phylogenetic trees were constructed using the neighbor-joining (NJ) algorithm in MEGA11.0 software.

### 2.3. Vector Construction and Agrobacterium-Mediated Genetic Transformation

#### 2.3.1. Construction of *VvALDH* Gene Overexpression and Silencing Vectors

**Figure 2:**
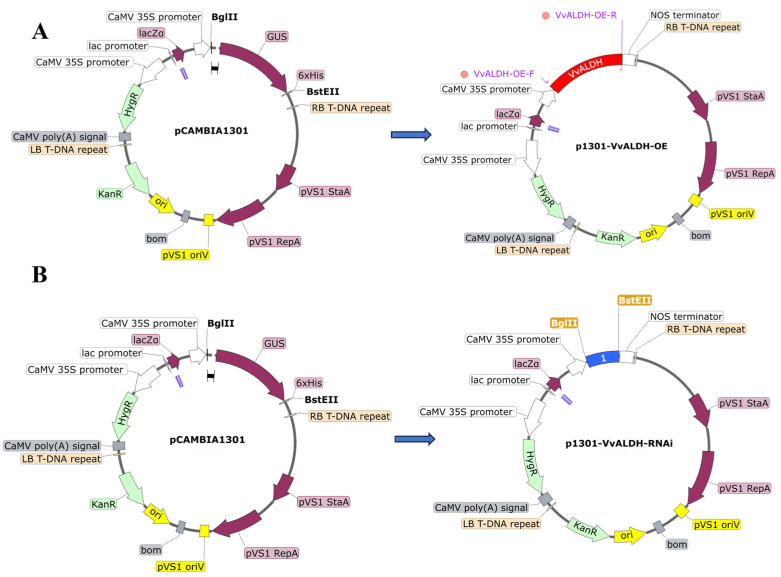
Construction of expression vectors. (**A**) Construction process of the overexpression vector; (**B**) Construction process of the RNAi vector. Notes: Red fragment indicates the cDNA sequence of the *VvALDH* gene; Blue fragment indicates the hairpin structure sequence.

The parent pCAMBIA-1301 vector was double-digested with *Bgl*II and *Bst*EII. The cDNA sequence of *VvALDH* (highlighted in red) was ligated into the vector by homologous recombination to generate the overexpression vector pCAMBIA-1301-*VvALDH*-OE (Figure 2A). The RNAi fragment of *VvALDH* was designed from the 13th exon (1801–1988 bp), using the 3rd intron (252–363 bp) as the loop. The parent pCAMBIA-1301 vector was double-digested, and the designed hairpin structure sequence (highlighted in blue) was ligated into the vector by homologous recombination to obtain the silencing vector pCAMBIA-1301-*VvALDH*-RNAi (Figure 2B). The vectors were synthesized by Beijing Qingke Company and verified by restriction enzyme digestion and sequencing.

#### 2.3.2. Establishment of an *Agrobacterium*-Mediated Transformation System for *V. volvacea*

Sensitivity test of *V. volvacea* mycelia to hygromycin (Hyg): Strain V9715 was inoculated onto PDA solid medium supplemented with 200 μg/mL ceftiofur sodium, containing Hyg at gradient concentrations (0, 10, 20, 30, 40, 50, 60, 70, 80, and 90 µg/mL). The cultures were statically incubated at 32 °C [25], and mycelial growth rate was measured using the cross method to assess the sensitivity of *V. volvacea* to Hyg.

Based on and modified from the transformation system for macrofungi [26], recombinant vectors were transferred into *A. tumefaciens* GV3101 following the instructions for competent cells. Single colonies were picked for expanded culture, and positive clones were verified by colony PCR using Hyg primers. The co-cultured mycelial blocks were transferred to IM medium and cultured at 28 °C for 3–5 days.

Screening of positive transformants: Mycelial blocks were transferred to PDA solid medium containing 40 μg/mL Hyg and 200 μg/mL Cef, and incubated at 32 °C for primary screening. Resistant mycelia were subsequently transferred to PDA solid medium (50 μg/mL Hyg) for subculture; mycelial blocks that continued to grow were identified as positive transformants.

#### 2.3.3. Verification of Transformants

Stability verification: Mycelia growing on resistant medium were subcultured on non-resistant PDA medium for 4 generations, and stable transformants were retained. PCR amplification verification: gDNA was extracted from stably inherited transformants, and the Hyg screening gene was amplified by PCR using Hyg-F and Hyg-R primers for identification. qRT-PCR verification: β-tubulin (*β-TUB*) was used as the reference gene. The 20 μL reaction system was configured, with the amplification program as follows: 95 °C for 5 min (1 cycle); 95 °C for 10 s, 60 °C for 0 s (40 cycles). A melting curve program was also included: 95 °C for 15 s, 60 °C for 60 s, 95 °C for 15 s. Signal detection was performed to analyze the transcription levels of the *VvALDH* gene in transformants.

### 2.4. Determination of Transformant Traits

#### 2.4.1. Determination of Mycelial Characteristics and Growth Rate

Solid culture mycelial characteristics: Overexpression transformants OE13, OE14, OE19, OE20, silenced transformants RNAi5, RNAi6, RNAi7, and the wild-type (WT) strain were cultured on solid media to assess mycelial growth rate and morphology. Mycelial morphology and biomass were also determined after submerged culture [27]. Mycelial growth rate measurement: Each culture bottle contained 250 g of medium (75% cottonseed hulls, 24% bran, 1% lime; water content 65%), filled to a uniform depth of 7 cm from the bottle mouth. Five mycelial plugs (5 mm diameter) were inoculated per bottle, with five replicate bottles per strain. Cultures were incubated at 32 °C in a constant-temperature incubator. Photographs were taken every 5 days to monitor downward mycelial growth.

#### 2.4.2. Determination of Primordium Indices and Fruiting Body Yield of Transformants

Transformants OE20, RNAi6, and the wild-type (WT) strain were inoculated onto cottonseed hull medium, with 15 mycelial plugs (4 mm in diameter) per bottle and 15 replicates per strain. Samples were collected at the primordium stage. The contents of key intermediates in the IAA biosynthesis pathway associated with the *VvALDH* gene were determined by high-performance liquid chromatography (HPLC), and ALDH activity was measured using an ALDH activity assay kit (Beijing Boxbio Science & Technology Co., Ltd., Beijing, China).

For fruiting body yield determination: After the mycelia had fully colonized the substrate, fruiting was induced in a 715 m^2^ mushroom house; each compartment had an area of 0.6 m × 1.2 m = 0.72 m^2^. The substrate consisted of a 1:1 mixture of spent substrates from *Lyophyllum decastes* and *Pleurotus eryngii*, with a 70% water content [28]. Each compartment contained 40 kg of wet substrate, with WT as the control. The time of primordium emergence was recorded, and the yield, number, and individual mushroom weight were measured.

#### 2.4.3. Detection of Key Genes in the IAA Synthesis Pathway of *V. volvacea*

Based on transcriptomic data, primers were designed for key genes in three IAA synthesis pathways involving ALDH: gene_7092 (tryptamine oxidase), gene_2093 (tryptophan side-chain oxidase), and gene_396 (indolepyruvate decarboxylase). PCR amplification and agarose gel detection were performed using fresh WT primordium-stage samples, following the method described in Section 2.2.

### 2.5. Data Analysis

Primers were designed using Primer Premier 5.0; sequencing results were assembled and edited with BioEdit 7.2.2; sequence alignment was performed and vector maps were constructed using SnapGene 6.0.2; and data analysis and graphing were conducted using Excel 2021 and Prism 10.

## 3. Results

### 3.1. Cloning and Bioinformatics Analysis of VvALDH

#### 3.1.1. Cloning of *VvALDH* Gene

**Figure 3:**
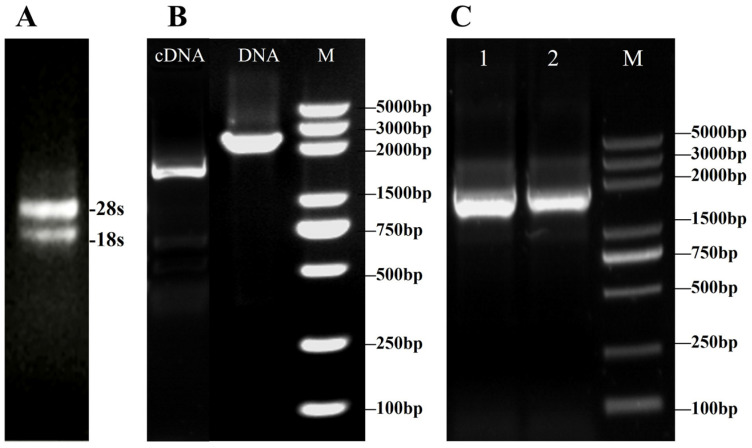
Cloning of the *VvALDH* gene. (**A**) RNA quality assay; (**B**) PCR amplification of *VvALDH* cDNA and genomic DNA, M: Marker DL 5000; (**C**) Colony PCR verification of the cloning vector, lanes 1 and 2 represent selected single colonies.

Total RNA was extracted from *V. volvacea*, and agarose gel electrophoresis revealed clear 28S and 18S rRNA bands, with the 28S band intensity being approximately twice that of the 18S band (Figure 3A), indicating good RNA integrity. RNA purity and concentration, as determined by a micro-spectrophotometer, met the experimental requirements. Using cDNA and gDNA as templates, PCR products were analyzed on 1% agarose gel electrophoresis and matched the predicted sizes (Figure 3B). These results confirmed successful ligation of the cloned gene into the pMD18-T vector. Sequence alignment with transcriptomic data showed 99% consistency, with the cDNA sequence being 1509 bp in length and the gDNA sequence measuring 2427 bp.

#### 3.1.2. Bioinformatics Analysis of *VvALDH* Gene

**Figure 4:**
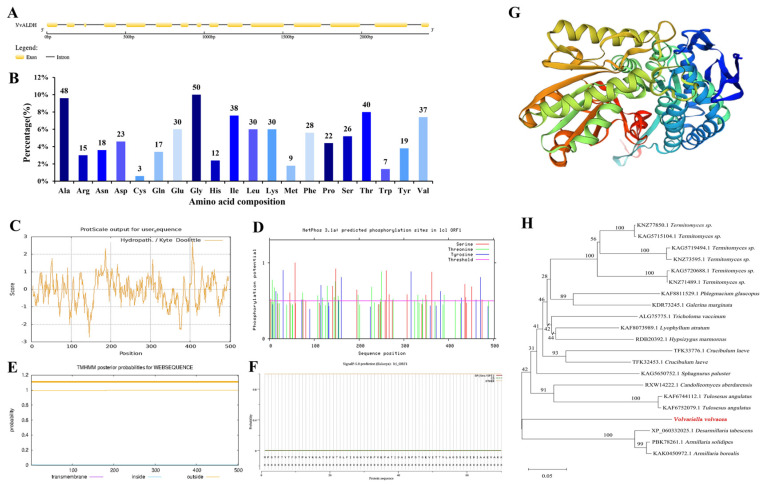
Bioinformatics analysis of *VvALDH*. (**A**) Gene structure of the *VvALDH*; (**B**) Amino acid sequence; (**C**) Protein hydrophobicity analysis; (**D**) Phosphorylation site prediction; (**E**) Protein transmembrane region analysis; (**F**) Signal peptide prediction; (**G**) Protein tertiary structure prediction; (**H**) Phylogenetic tree of *VvALDH*.

The *VvALDH* gene has a full length of 2427 bp, containing 15 exons, 14 introns, and an ORF of 1509 bp (Figure 4A). Predictions of protein family and conserved domains revealed that *VvALDH* belongs to the aldehyde dehydrogenase family (Architecture ID: 10162896), exhibiting similarity to NAD/NADP/NAD(P)-dependent aldehyde dehydrogenases and predicted to use NADP^+^ as its primary coenzyme. *VvALDH* encodes 502 amino acids (Figure 4B), with glycine (Gly) being the most abundant. Its physicochemical properties are as follows: molecular weight, 54,691.20 Da; theoretical isoelectric point (pI), 5.88; molecular formula, C_2480_H_3819_N_643_O_729_S_12_; total number of atoms, 7683. The instability index is 26.92, indicating that the protein is stable. The grand average of hydropathicity (GRAVY) is −0.072 (range: −2.733 to 2.767), classifying it as a hydrophobic protein (Figure 4C). Phosphorylation primarily occurs at serine (Ser), threonine (Thr), and tyrosine (Tyr) residues (Figure 4D). The protein lacks transmembrane domains and signal peptides (Figure 4E,F), with all 1–502 amino acids predicted to be localized in the cytoplasm rather than extracellularly. Its secondary structure comprises 32.87% α-helices, 23.71% extended strands, 11.95% turns, and 31.47% random coils. The tertiary structure model has a GMQE score of 0.98 and exhibits 71.51% sequence similarity, covering amino acids 1–502 (Figure 4G), indicating high prediction reliability. The amino acid sequence of *VvALDH* shares high similarity and homology KAF8647366.1 and KAF8647313.1 (from *V. volvacea*). Additionally, it shows notably high homology to sequences from *Armillaria borealis* and *Armillaria solidipes* (Figure 4H).

### 3.2. Construction of Overexpression and RNAi Vectors for VvALDH in V. volvacea and Establishment of Genetic Transformation System

#### 3.2.1. Validation of *VvALDH* Overexpression and Silencing Vectors, and Hyg Sensitivity Assay

**Figure 5:**
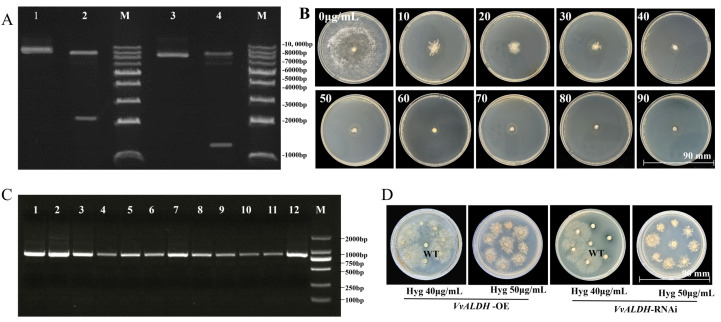
Validation of *VvALDH* overexpression and RNAi vectors, and hygromycin sensitivity assay. (**A**) Restriction digestion verification of recombinant vectors; (**B**) Hygromycin susceptibility test of *V. volvacea* mycelia; (**C**) PCR detection of the Hyg gene in *Agrobacterium tumefaciens* transformants; (**D**) Screening results of transformants on hygromycin medium. Notes: (**A**) Lane 1: linearized pCAMBIA-1301; Lane 2: pCAMBIA-1301-*VvALDH*-OE; Lane 3: linearized pCAMBIA-1301; Lane 4: pCAMBIA-1301-*VvALDH*-RNAi; M: DL 10,000 DNA Marker. (**C**) Lanes 1–12: PCR products of individual colonies.

The *VvALDH* gene was inserted into the GUS gene locus of the pCAMBIA-1301 vector by homologous recombination, generating pCAMBIA-1301-*VvALDH*-OE (overexpression vector) and pCAMBIA-1301-*VvALDH*-RNAi (silencing vector), both of which harbored the Hyg resistance marker gene. Double restriction enzyme digestion of the constructed vectors revealed clear bands of the expected sizes (Figure 5A), confirming successful vector construction. Hyg concentration gradient assays (Figure 5B) demonstrated dose-dependent inhibition of mycelial growth. At 40 μg/mL Hyg, mycelial germination rate decreased significantly; at 50 μg/mL, mycelial growth was completely inhibited while maintaining cell viability, indicating high sensitivity of *V. volvacea* mycelia to Hyg. Thus, 40 μg/mL and 50 μg/mL were used as the concentrations for primary and secondary selection, respectively. Following transformation of the constructed vectors into *Agrobacterium* GV3101, single colonies were selected from LB selective plates containing kanamycin (Kan) and rifampicin (Rif). PCR amplification of the Hyg gene (845 bp) showed that all 12 selected colonies yielded the expected amplicons (Figure 5C), confirming successful vector transformation into *Agrobacterium*. After co-cultivation, mycelial plugs were inoculated onto selective medium containing 40 μg/mL Hyg. Approximately 4 days later, mycelial germination was observed in transformants but not in the WT strain. Mycelia that germinated during primary screening were transferred to fresh PDA medium containing 50 μg/mL Hyg for secondary screening. After 10 days, partial mycelial germination was observed in transformants, whereas no germination occurred in the WT (Figure 5D).

#### 3.2.2. Quantitative Validation of *VvALDH* Overexpression and RNAi Transformants

**Figure 6:**
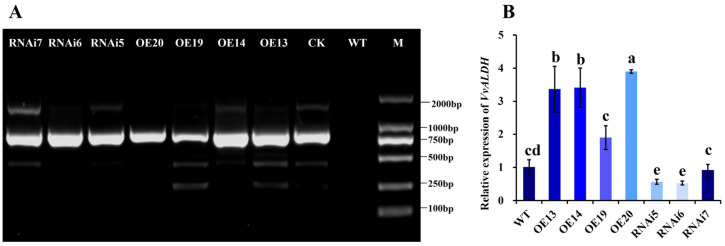
Quantitative validation of *VvALDH* overexpression and RNAi transformants. (**A**) PCR detection of the Hyg gene in transformants; (**B**) qRT-PCR analysis of *VvALDH* expression. Notes: WT: wild-type strain; CK: empty vector control; RNAi5–7: RNAi transformants; OE13, 14, 19, 20: overexpression transformants; M: DL2000 Marker. Different letters (a–e) indicate significant differences (*p* < 0.05).

After 4 generations of subculture, mycelia were re-inoculated onto selective medium containing 50 μg/mL Hyg, and stable transformants were selected for further analysis. Genomic DNA was extracted from these transformants for PCR amplification of the Hyg gene sequence (Figure 6A), resulting in the identification of 4 overexpression transformants and 3 RNAi transformants. The WT strain showed no amplification band, whereas all transformants exhibited bands of the expected size (845 bp), consistent with the plasmid control (as expected). qRT-PCR was used to determine the relative expression of *VvALDH* at the mRNA level (Figure 6B). Overexpression transformants OE13, OE14, and OE20 exhibited 3.37-fold, 3.41-fold, and 3.9-fold upregulation, respectively, with expression levels significantly higher than that in the WT. In contrast, OE19 showed a 1.90-fold upregulation, with no statistically significant difference from the WT. For RNAi transformants, RNAi5, RNAi6, and RNAi7 displayed interference efficiencies of 44.12%, 48.04%, and 10.78%, respectively. Notably, RNAi7 showed no significant difference from the WT, indicating low silencing efficiency.

#### 3.2.3. Effects of *VvALDH* on Mycelial Morphology and Growth Rate

**Figure 7:**
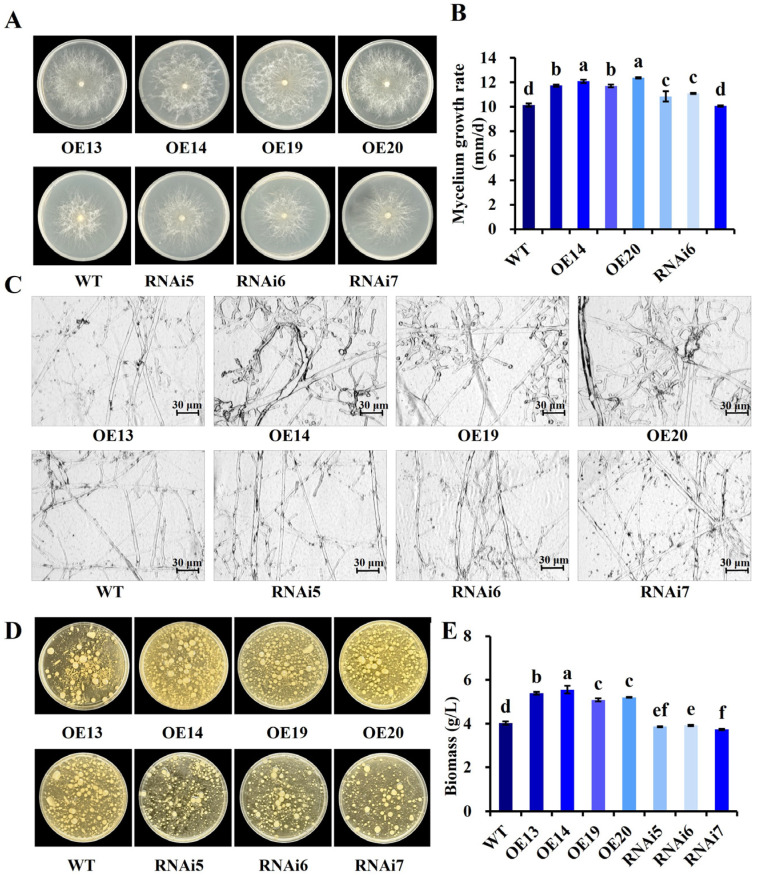
Effects of *VvALDH* on mycelial morphology and growth. (**A**) Colony morphology of transformants on plates; (**B**) Mycelial growth rate on plates; (**C**) Microscopic morphology of transformant hyphae; (**D**) Growth of transformants in liquid culture; (**E**) Biomass of transformants. Notes: In Figure 7B,E different letters (a–f) indicate significant differences (*p* < 0.05).

The mycelial growth rate of the 4 overexpression transformants were significantly higher than WT (*p* < 0.05), with OE20 exhibiting the fastest growth rate (12.37 ± 0.05 mm/d) and denser, whiter mycelia (Figure 7A). For RNAi transformants, no significant differences were observed compared to the WT control; among them, RNAi5 and RNAi6 showed higher growth rates than the WT, while RNAi7 showed a lower rate. (Figure 7B). Microscopic observation of overexpression transformant mycelia (Figure 7C) revealed increased branching and thicker hyphae (except OE13) compared to the WT, whereas RNAi transformants showed morphology similar to the WT control. Analysis of mycelial morphology, density and biomass (Figure 7D,E) after 5 days of culture indicated that overexpression transformants had significantly higher biomass than the WT. Specifically, OE14 exhibited the highest biomass (5.56 g/L), representing a 37.97% increase compared to the WT. In contrast, RNAi transformants had significantly lower biomass than the WT, which was consistent with the results from solid medium culture.

#### 3.2.4. Effect of *VvALDH* on Mycelial Growth Rate in Growth Substrate

**Figure 8:**
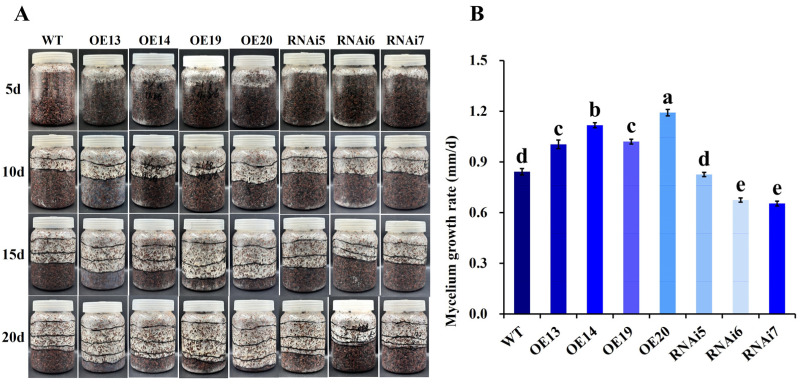
Effect of *VvALDH* on mycelial growth in substrate. (**A**) Mycelial growth of transformants; (**B**) Mycelial growth rates of transformants. Note: In Figure 8B, different letters (a–e) indicate significant differences (*p* < 0.05).

After inoculation, transformants were incubated at 32 °C in the dark for 20 days. Mycelial growth was monitored by measuring and photographing at 5-day intervals (Figure 8A). The mycelial growth rates of all overexpression transformants were significantly higher than that of the WT (*p* < 0.05). Notably, the OE20 transformant exhibited the fastest growth rate (1.19 mm/d), which was 1.42-fold that of the WT, with pure white mycelia, followed by OE14 (Figure 8B). In contrast, the growth rates of RNAi6 and RNAi7 were significantly lower than that of the WT, indicating effective gene silencing, while RNAi5 showed no significant difference in growth trend compared to the WT.

### 3.3. Quantitative Analysis of VvALDH Expression During Primordium Formation and Its Impact on IAA Biosynthesis

**Figure 9:**
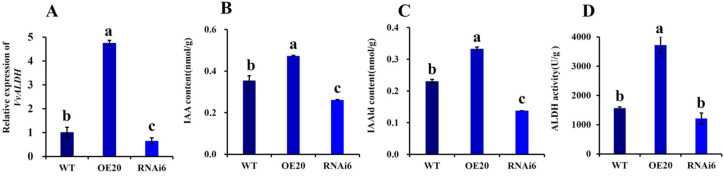
Quantitative analysis of *VvALDH* expression during primordium formation and its impact on IAA biosynthesis. (**A**) Relative quantification of the *VvALDH* gene; (**B**) IAA (indole-3-acetic acid) content; (**C**) IAAld (indole-3-acetaldehyde) content; (**D**) ALDH (aldehyde dehydrogenase) content. Note: letters a–c, respectively, represent the degree of significant difference (*p* < 0.05).

Based on mycelial stage indices and gene quantification results, the overexpression transformant OE20, RNAi transformant RNAi6, and WT were selected for fruiting experiments. Samples were collected on day 8 after sowing, when primordia appeared, to determine the relative expression of *VvALDH* (Figure 9A). Compared to the WT, OE20 exhibited a 4.76-fold upregulation, while RNAi6 showed a 35% decrease in expression. After sampling primordia, the contents of key intermediates in the IAA synthesis pathway and ALDH enzymatic activity were measured. All five intermediate products in OE20 were significantly higher than those in the WT and RNAi6. Specifically, compared to the WT, OE20 had a 34.29% higher IAA content (Figure 9B), 43.48% higher IAAld content (Figure 9C), and 137.26% higher ALDH activity (Figure 9D) while maintaining cell viability, indicating high sensitivity of *V. volvacea* mycelia to Hyg. Thus, 40 μg/mL and 50 μg/mL were used as the concentrations for primary and secondary selection.

### 3.4. Effect of VvALDH on Yield and Productivity

**Figure 10:**
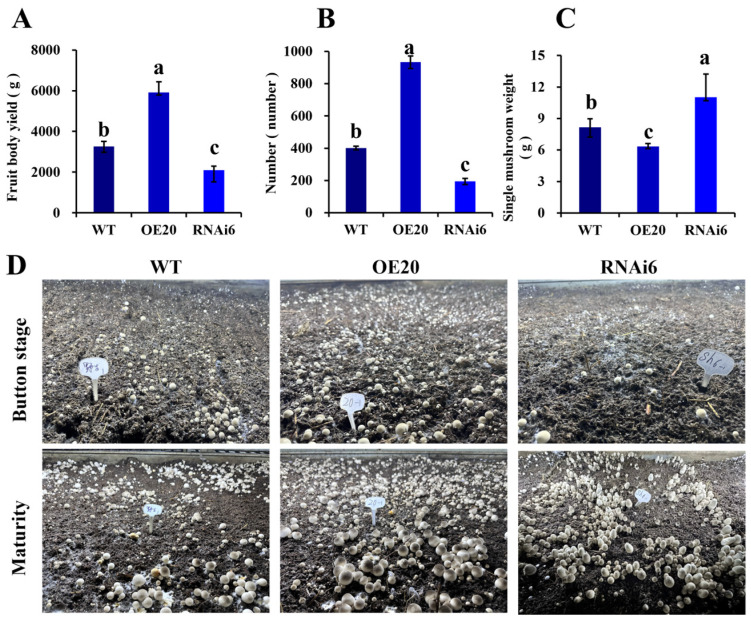
Effect of *VvALDH* on yield and productivity. (**A**) Total yield of transformants; (**B**) Number of fruiting bodies; (**C**) Average weight of single fruiting bodies; (**D**) Fruiting body development at different stages. Note: Different letters (a–c) indicate significant differences (*p* < 0.05).

Compared to the WT, OE20 showed an 81.57% increase in yield and produced 133.11% more fruiting bodies. These results indicate that overexpression of *VvALDH* significantly improved the yield and number of fruiting bodies in *V. volvacea*, while reducing the average individual fruiting body weight and promoting primordium differentiation. Fruiting bodies were harvested starting from day 10 after sowing, with yield and number recorded over 3 consecutive days. The overexpression transformant OE20 exhibited significantly higher total yield and number of fruiting bodies compared to WT (Figure 10A,B), with substantially more button-stage and mature fruiting bodies (Figure 10D). Compared to the WT, OE20 showed 81.57% higher yield and 133.11% more fruiting bodies, respectively. These results indicate that overexpression of *VvALDH* significantly improved the yield and number of fruiting bodies in *V. volvacea*, reduced individual fruiting body weight, and promoted primordium differentiation. In contrast, the yield and number of fruiting bodies of RNAi6 were significantly lower than those of the WT, with decreases of 35.54% and 51.66%, respectively. Meanwhile, the individual fruiting body weight of RNAi6 was significantly higher than that of both the WT and OE20 (Figure 10C). Additionally, the fruiting performance of RNAi6 at the button and mature stages was significantly weaker than that of the WT.

### 3.5. Detection of Key Genes in the IAA Synthesis Pathway of and Content of Intermediate Products in V. volvacea

**Figure 11:**
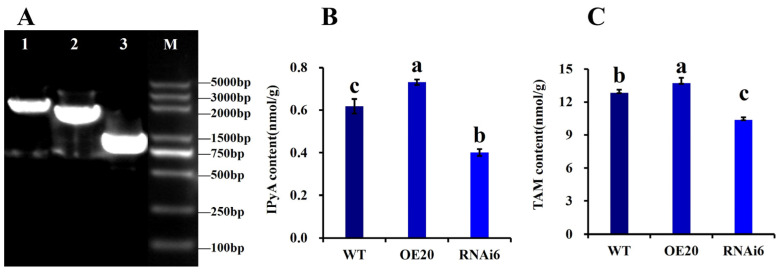
Detection of key genes in IAA synthesis pathway of and content of intermediate products in *V. volvacea*. (**A**) PCR detection; (**B**) IPyA (indole-3-pyruvate) content; (**C**) TAM (indole-3-acetamide) content. Note: Lane 1: gene_7092 (tryptamine oxidase); Lane 2: gene_396 (indole pyruvate decarboxylase); Lane 3: gene_2093 (tryptophan side chain oxidase); M: Marker; different letters (a–c) indicate significant differences (*p* < 0.05).

During the primordium stage, PCR amplification and detection of key genes in the three ALDH-involved IAA synthesis pathways revealed that the amplicon of gene_7092 (tryptamine oxidase) matched the predicted length of 2341 bp, that of gene_396 (indole-pyruvate decarboxylase) matched 1821 bp, and that of gene_2093 (tryptophan side-chain oxidase) matched 816 bp (Figure 11A). These results confirm that all three pathways are involved in IAA synthesis in *V. volvacea*, with IAAld being synthesized first and then converted by ALDH into IAA. The contents of IPyA and TAM, the intermediates of the two pathways involved in ALDH. Compared to WT, there is 17.74% more IPyA (Figure 11B) and 6.61% more TAM, with significantly higher levels of the relevant intermediates in the IPyA and TAM pathways. In contrast, RNAi6 showed a 25.71% lower IAA content, 39.13% lower IAAld content, 22.73% lower ALDH activity, 33.87% lower IPyA content, and 19.30% lower TAM content compared to the WT (Figure 11C).

## 4. Discussion

Based on previous transcriptome analysis of key genes in the IAA synthesis pathway of *V. volvacea*, combined with differential expression analysis and literature evidence. The *VvALDH* gene was selected for cloning and sequence characterization, followed by promoter and evolutionary analysis. This gene encodes a key enzyme that catalyzes the conversion of IAAld to IAA. The *VvALDH* gene of *V. volvacea* encodes 502 amino acids. It contains 15 exons and 14 introns, and undergoes phosphorylation primarily at serine and threonine residues; glycine is the most abundant amino acid. The full-length *VvALDH* gene is 2427 bp, with a CDS of 1509 bp and NADP^+^ as the coenzyme, a structure that shows strong similarity to the six *Glaldh* genes identified in *Ganoderma lucidum* [13]. Chen [29] demonstrated that the tryptamine pathway involves three key enzymes: AADC, MAO, and ALDH. Among these, ALDH exhibits high expression, while AADC and MAO, despite their low expression, act as rate-limiting enzymes in the first two steps of the pathway.

*Agrobacterium*-mediated transformation, which enables stable single-copy integration, has become a widely used technique for fungal genetic manipulation [30]. Although most commercial vectors were originally designed for plants, modification of the pCAMBIA-1301 vector allowed us to construct effective silencing and overexpression vectors for *V. volvacea*. Similar strategies have been successfully applied in edible fungi, such as *Auricularia auricula* [23] and *Phellinus baumii* [26]. Zhang [31] employed ATMT to overexpress the cellulase gene in *Auricularia auricula*, successfully generating strains with a 40% increase in cellulose degradation efficiency. Research on *Agrobacterium*-mediated fungal transformation remains relatively limited, with recipient materials primarily being protoplasts, mycelia, or spores. However, protoplast-based methods are technically demanding, inefficient, and dependent on enzymatic digestion quality [21]. In this study, we used mycelia as transformation recipients and employed hygromycin resistance for selection, providing a simple and reproducible system for generating transformants. Interestingly, we observed that 50 μg/mL hygromycin was sufficient to completely inhibit mycelial growth, which differs slightly from the 40 μg/mL reported by Meng [25]. Such differences may reflect strain-specific traits, recipient types, or medium formulations, emphasizing the importance of optimizing screening conditions for each experimental system.

Four overexpression transformants and three RNAi transformants were screened to determine mycelial characteristics, key intermediates in the IAA synthesis pathway, and aldehyde dehydrogenase activity. Overexpression lines exhibited markedly enhanced mycelial growth rates, improved hyphal morphology, and greater biomass compared with the WT, suggesting that *VvALDH* overexpression increases mycelial vitality and promotes metabolic activity. This observation is consistent with the findings of Hou et al. [32], who reported that adding 0.70 g/L NaAc during submerged culture maximized biomass and DNA content, indicating that NaAc enhances ALDH synthesis and thereby stimulates mycelial proliferation. By contrast, the RNAi5 line showed no significant differences from the control in biomass, growth rate, or hyphal extension. Interestingly, gene expression quantification at the mycelial stage revealed no significant differences in OE20 and RNAi7 compared to WT, which may be attributed to unstable expression or false positives during selection. Therefore, OE20 and RNAi6—both exhibiting consistent changes in gene expression and phenotype—were chosen for subsequent fruiting experiments. During the primordium stage, OE20 displayed significantly upregulated *VvALDH* expression, elevated IAA content, and improved yield, whereas RNAi6 showed suppressed expression, reduced IAA content, and diminished yield. These findings are in agreement with Liu [33], who demonstrated that single-gene knockout of the IAAld dehydrogenase gene (*ald2*) and the nitrilase gene (*nit2*) in *Trichoderma harzianum* markedly decreased IAA content. Together, these results provide strong evidence that *VvALDH* directly regulates IAA biosynthesis, primordium differentiation, and yield formation in *V. volvacea*.

In *Pleurotus ostreatus*, the aldehyde dehydrogenase gene *PoALDH1* exhibits significant temporal expression differences during fruiting body development, and increased exogenous IAA concentration inhibits mycelial growth rate [34]. This aligns with our findings that *VvALDH* overexpression enhances IAA biosynthesis during the primordium stage, highlighting its role as a regulatory node in fungal IAA metabolism. Furthermore, our results support previous reports that ALDH catalyzes the terminal reaction of the IPyA pathway. For instance, the ectomycorrhizal-specific gene *TfAld1* in *Tricholoma fulvum* is strongly induced by IAAld and highly expressed [35]. Notably, we observed that the inhibitory effects of *VvALDH* silencing on mycelial growth and IAA production were less pronounced than the stimulatory effects of overexpression, suggesting potential functional redundancy among ALDH family members. Similar phenomena have been reported in *Neurospora crassa*, where multiple ALDH genes act synergistically in the IAA biosynthetic pathway, and mutations in key genes significantly impair conidial and mycelial development [17]. IAA levels also fluctuate dynamically during the developmental stages of *Ganoderma lucidum*, where three key enzymes—DDC, AOC1, and ALDH—have been identified, indicating the coexistence of both IPyA and TAM pathways [36]. As multifunctional housekeeping enzymes, ALDHs not only detoxify reactive aldehydes by converting them into corresponding carboxylic acids, but also participate in diverse metabolic processes including polyamine catabolism, ethanol metabolism, and xenobiotic degradation [37].

Furthermore, the IAA content of *V. volvacea* exhibits dynamic changes across different developmental stages, indicating its pivotal role in coordinating fungal growth and morphogenesis [29]. Despite these observations, the precise regulatory networks and molecular mechanisms underlying IAA biosynthesis and signaling in edible fungi remain largely unexplored. Future investigations could integrate high-resolution HPLC profiling of intermediates from distinct IAA biosynthetic pathways with targeted assays of key enzyme activities throughout the developmental stages of *V. volvacea*. Ultimately, this study highlights *VvALDH* as a central regulator of fungal IAA metabolism and offers a foundation for future biotechnological interventions aimed at enhancing the growth and productivity of edible mushrooms.

## 5. Conclusions

In this study, we successfully cloned and characterized the *VvALDH* gene from *V. volvacea* and confirmed that it encodes a conserved aldehyde dehydrogenase family protein. Expression profiling revealed that *VvALDH* is significantly upregulated during primordium formation and closely associated with IAA biosynthesis. Functional verification using *Agrobacterium*-mediated transformation demonstrated that *VvALDH* overexpression enhanced mycelial biomass, accelerated primordium differentiation, and markedly increased fruiting body yield, whereas RNAi-mediated silencing produced the opposite effects. Furthermore, metabolic analyses indicated that *VvALDH* regulates IAA synthesis through the IPyA, TAM, and TSO pathways, highlighting its central role in auxin metabolism. Collectively, these findings provide the first functional evidence that *VvALDH* positively regulates primordium initiation and yield formation in *V. volvacea* by modulating IAA biosynthesis. This study not only advances our understanding of the molecular mechanisms underlying fruiting body development in edible fungi but also offers a potential genetic target for improving yield and productivity in mushroom cultivation.

## Figures and Tables

**Table 1:** Information of primers used for *VvALDH* gene cloning and functional verification.

Primer Name	Primer Sequence (5′-3′)	Application
*VvALDH*-1F	CCCTCAAACCATGCCTTCTACC	Gene sequence amplification
*VvALDH*-1R	CGCACAAAAGGGCGATTACAA	
M13F-47	CGCCAGGGTTTTCCCAGTCACGAC	Cloning verification
M13R-48 (RV-M)	AGCGGATAACAATTTCACACAGGA	
*VvALDH*-OE-F	TGACCATGGTAGATCTATGCCTTCTACCTTCACATACACCTT	Overexpression primer
*VvALDH*-OE-R	ATTCGAGCTGGTCACCTTACAACTTCATACCAAGGTTAAGATGGACG	
*VvALDH*-RNAi-1F	TCCTCTATATAAGGAAGTTCATTTCATTTGGAGAGAACACGGGGGACTCTTGACCATGGTA	RNAi primer
*VvALDH*-RNAi-1R	CTTAAGAAACTTTATTGCCAAATGTTTGAACGATCGGGGAAATTCGAGCTGGTCACCCGTAT	
Hyg-F	ATTTGTGTACGCCCGACAGT	Verify the primer
Hyg-R	CTCTCGGAGGGCGAAGAATC	
*β-TUB*-F	TCAGGCAGGTGTCCAGAT	Internal reference primer
*β-TUB*-R	TCGGAGAAGAAAGTGCTG	
*VvALDH*-qpcr-F	CGGATATTTGTGCAAGAGGG	Real-time quantitative PCR
*VvALDH*-qpcr-R	GTAGTTGATGCGAACGGGTC	
*MAO*-F	CTTGTTTCGTGGCTCTT	Gene sequence amplification
*MAO*-R	AGGCTGCTATGTCCTCTAC	
*TSO*-F	GCAAGGCGGTGTTCATA	
*TSO*-R	AGCCATAGCCAATCCAG	
*IPDC*-F	CTGGGTTTCTTGGTGAGT	
*IPDC*-R	AGGAATACAATCGGCTTT	

## Data Availability

The original contributions presented in this study are included in the article. Further inquiries can be directed to the corresponding author.
